# AI-based diagnosis of nuclear cataract from slit-lamp videos

**DOI:** 10.1038/s41598-023-49563-7

**Published:** 2023-12-12

**Authors:** Eisuke Shimizu, Makoto Tanji, Shintato Nakayama, Toshiki Ishikawa, Naomichi Agata, Ryota Yokoiwa, Hiroki Nishimura, Rohan Jeetendra Khemlani, Shinri Sato, Akiko Hanyuda, Yasunori Sato

**Affiliations:** 1 OUI Inc., Tokyo, Japan; 2 Department of Ophthalmology, Keio University School of Medicine, Tokyo, Japan; 3 Yokohama Keiai Eye Clinic, Yokohama, Japan; 4 Department of Preventive Medicine and Public Health, School of Medicine, Keio University, Tokyo, Japan

**Keywords:** Eye diseases, Mechanical engineering

## Abstract

In ophthalmology, the availability of many fundus photographs and optical coherence tomography images has spurred consideration of using artificial intelligence (AI) for diagnosing retinal and optic nerve disorders. However, AI application for diagnosing anterior segment eye conditions remains unfeasible due to limited standardized images and analysis models. We addressed this limitation by augmenting the quantity of standardized optical images using a video-recordable slit-lamp device. We then investigated whether our proposed machine learning (ML) AI algorithm could accurately diagnose cataracts from videos recorded with this device. We collected 206,574 cataract frames from 1812 cataract eye videos. Ophthalmologists graded the nuclear cataracts (NUCs) using the cataract grading scale of the World Health Organization. These gradings were used to train and validate an ML algorithm. A validation dataset was used to compare the NUC diagnosis and grading of AI and ophthalmologists. The results of individual cataract gradings were: NUC 0: area under the curve (AUC) = 0.967; NUC 1: AUC = 0.928; NUC 2: AUC = 0.923; and NUC 3: AUC = 0.949. Our ML-based cataract diagnostic model achieved performance comparable to a conventional device, presenting a promising and accurate auto diagnostic AI tool.

## Introduction

Blindness and visual impairment are increasingly being reported worldwide, with estimates suggesting that cases of visual impairment will increase to 115 million by 2050^1^. Cataracts are the leading cause of blindness, particularly in developing countries^2^, and contribute to 52.63 disability-adjusted life years (DALYs) per million indivduals^3^. Moreover, the DALYs due to cataracts in individuals over 65 years of age is estimated to match or surpass those of tuberculosis and acquired immune deficiency syndrome^4,5^.

Cataracts are the most common age-related disease, typically treated through lens-replacement surgery^6^. Slit-lamp microscopy serves as the primary method for diagnosing cataracts^7^. However, because of the high prevalence of the disease and inadequate medical resources, especially in underdeveloped countries, cases of blindness caused by cataracts continue to rise^2,6,8^.

The feasibility of using artificial intelligence (AI) developed using machine learning (ML) algorithms has been studied extensively for screening and diagnosing retinal and optic nerve disorders. These algorithms primarily rely on widely accessible fundus photographs and optical coherence tomography images^9–14^.

However, only few studies have focused on the anterior segment of eyes^15–17^. Although an AI algorithm to diagnose anterior segment diseases using slit-lamp photographs and topography images has been proposed, inadequate big data and analytical strategies limit the development of diagnostic AI tools for assessing the anterior segment, including cataract diagnosis^18^. Consequently, anterior segment screening and diagnosis using AI remains underdeveloped.

Therefore, we proposed a novel solution by incorporating video recording during slit-lamp examinations to facilitate assessment through an ML algorithm. Videos are advantageous because: (1) they are an aggregation of multiple images; therefore, multiple images can be extracted for analysis, increasing the ease of the collection of large quantities of data, and (2) a video mimics clinical ophthalmology setting where an ophthalmologist performs diagnosis using consecutive images obtained using a slit-lamp microscope. Therefore, we used a recordable slit-lamp device to film cataract videos^19^. This study investigated whether the proposed ML method could diagnose nuclear cataracts (NUCs) by using a video filmed using a slit-lamp device. Furthermore, a grading system was used to classify NUCs.

## Results

### Dataset size

A total of 38,320 frames from 1042 videos were used for training, validation, and testing of ML models. Consequently, 11,243 frames from 444 videos with grade NUC 0, 12,241 frames from 289 videos with grade NUC 1, 12,637 frames from 280 videos with grade NUC 2, and 2199 frames from 54 videos with grade NUC 3 were used.

### Diagnostic performance according to the frames

**Figure 1:**
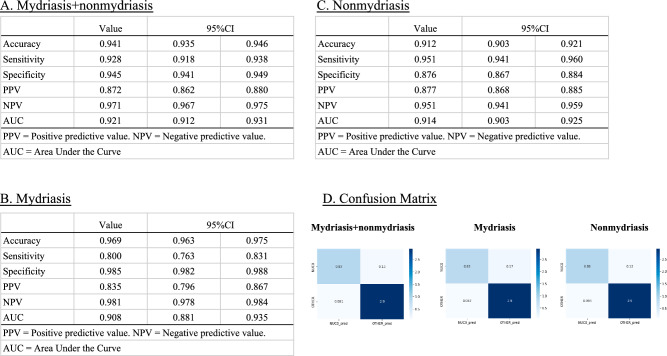
Diagnostic performance according to frames. Diagnostic performance of our machine learning model against ophthalmologist diagnosis according to frame. (**A**) Mydriasis + Non mydriasis: Accuracy: 0.941 (95% confidence interval [CI], 0.935–0.946); Sensitivity: 0.928 (95% CI, 0.918–0.938); Specificity: 0.945 (95% CI, 0.941–0.949); Positive predictive value (PPV): 0.872 (95% CI, 0.862–0.880); Negative predictive value (NPV): 0.971 (95% CI, 0.967–0.975); the area under the curve (AUC) for the receiver operating characteristic: 0.921 (95% CI, 0.912–0.931). (**B**) Mydriasis only: Accuracy: 0.969 (95% CI, 0.935–0.946); Sensitivity: 0.800 (95% CI, 0.763–0.831); Specificity: 0.985 (95% CI, 0.982–0.988); PPV: 0.835 (95% CI, 0.796–0.867); NPV: 0.981 (95% CI, 0.978–0.984); AUC: 0.908 (95% CI, 0.881–0.935). (**C**) Non mydriasis only: Accuracy: 0.912 (95% CI, 0.903–0.921); Sensitivity: 0.951 (95% CI, 0.941–0.960); Specificity: 0.876 (95% CI, 0.867–0.884); PPV: 0.877 (95% CI, 0.868–0.885); NPV: 0.951 (95% CI, 0.941–0.959); AUC: 0.914 (95% CI, 0.903–0.925). (**D**) Confusion matrices of mydriasis + Non mydriasis, mydriasis only, and Non mydriasis only.

The diagnostic performance of our model was compared with that of ophthalmologists using a validation dataset. The diagnostic performance of the model against the ophthalmologist accompanying each frame is as follows: Mydriasis + Non mydriasis frames: Accuracy: 0.941 (95% CI, 0.935–0.946); Sensitivity: 0.928 (95% CI, 0.918–0.938); Specificity: 0.945 (95% CI, 0.941–0.949); PPV: 0.872 (95% CI, 0.862–0.880); NPV: 0.971 (95% CI, 0.967–0.975); AUC: 0.921 (95% CI, 0.912–0.931). Mydriasis frames only: Accuracy: 0.969 (95% CI, 0.935–0.946); Sensitivity: 0.800 (95% CI, 0.763–0.831); Specificity: 0.985 (95% CI, 0.982–0.988); PPV: 0.835 (95% CI, 0.796–0.867); NPV: 0.981 (95% CI, 0.978–0.984); AUC: 0.908 (95% CI, 0.881–0.935). Non mydriasis frames only: Accuracy: 0.912 (95% CI, 0.903–0.921); Sensitivity: 0.951 (95% CI, 0.941–0.960); Specificity: 0.876 (95% CI, 0.867–0.884); PPV: 0.877 (95% CI, 0.868–0.885); NPV: 0.951 (95% CI, 0.941–0.959); AUC: 0.914 (95% CI, 0.903–0.925) (Fig. 1).

### Diagnostic performance according to videos

**Figure 2:**
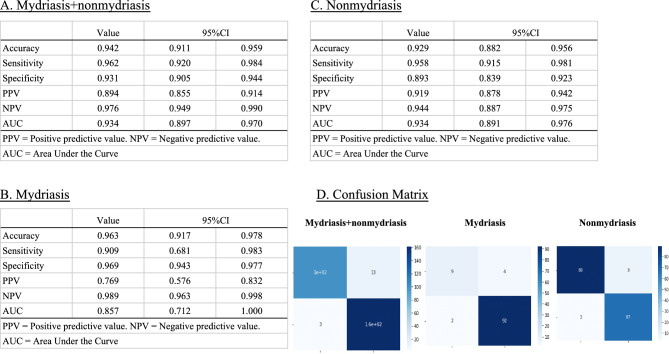
Diagnostic performance according to the videos. Diagnostic performance of our machine learning model against ophthalmologist diagnoses according to each video. (**A**) Mydriasis + Non mydriasis: Accuracy: 0.942 (95% confidence interval [CI], 0.911–0.959); Sensitivity: 0.962 (95% CI, 0.920–0.984); Specificity: 0.931 (95% CI, 0.905–0.944); PPV: 0.894 (95% CI, 0.855–0.914); NPV: 0.976 (95% CI, 0.949–0.990); the AUC for the receiver operating characteristic: 0.934 (95% CI, 0.897–0.970). (**B**) Mydriasis only: Accuracy: 0.963 (95% CI, 0.917–0.978); Sensitivity: 0.909 (95% CI, 0.681–0.983); Specificity: 0.969 (95% CI, 0.943–0.977); PPV: 0.769 (95% CI, 0.576–0.832); NPV: 0.989 (95% CI, 0.963–0.998); AUC: 0.857 (95% CI, 0.712–1.000). (**C**) Non mydriasis only: Accuracy: 0.929 (95% CI, 0.882–0.956); Sensitivity: 0.958 (95% CI, 0.915–0.981); Specificity: 0.893 (95% CI, 0.839–0.923); PPV: 0.919 (95% CI, 0.878–0.942); NPV: 0.944 (95% CI, 0.887–0.975); AUC: 0.934 (95% CI, 0.891–0.976). (**D**) Confusion matrices of mydriasis + Non mydriasis, mydriasis only, and Non mydriasis only.

The diagnostic performance of the model against the ophthalmologist accompanying each video was as follows: Mydriasis + Non mydriasis videos: Accuracy: 0.942 (95% CI, 0.911–0.959); Sensitivity: 0.962 (95% CI, 0.920–0.984); Specificity: 0.931 (95% CI, 0.905–0.944); PPV: 0.894 (95% CI, 0.855–0.914); NPV: 0.976 (95% CI, 0.949–0.990); AUC: 0.934 (95% CI, 0.897–0.970). Mydriasis videos only: Accuracy: 0.963 (95% CI, 0.917–0.978); Sensitivity: 0.909 (95% CI, 0.681–0.983); Specificity: 0.969 (95% CI, 0.943–0.977); PPV: 0.769 (95% CI, 0.576–0.832); NPV: 0.989 (95% CI, 0.963–0.998); AUC: 0.857 (95% CI, 0.712–1.000). Non mydriasis videos only: Accuracy: 0.929 (95% CI, 0.882–0.956); Sensitivity: 0.958 (95% CI, 0.915–0.981); Specificity: 0.893 (95% CI, 0.839–0.923); PPV: 0.919 (95% CI, 0.878–0.942); NPV: 0.944 (95% CI, 0.887–0.975); AUC: 0.934 (95% CI, 0.891–0.976) (Fig. 2).

### Diagnostic performance by each severity grade

**Figure 3:**
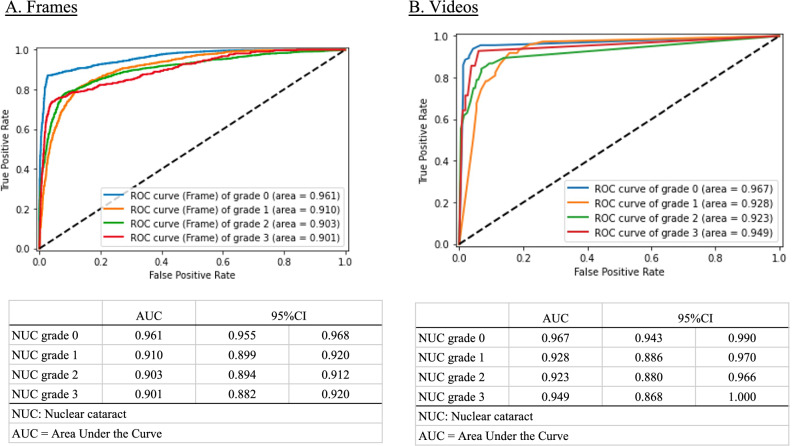
Diagnostic performance of our machine learning model by each severity grade against the performance of an ophthalmologist. (**A**) According to each frame: nuclear cataract (NUC) grade 0: AUC, 0.961 (95% CI, 0.955–0.968); NUC grade 1: AUC, 0.910 (95% CI, 0.899–0.920); NUC grade 2: AUC, 0.903 (95% CI, 0.894–0.912); NUC grade 3: AUC, 0.901 (95% CI, 0.882–0.920). (**B**) According to each eye: NUC grade 0: AUC, 0.967 (95% CI, 0.943–0.990); NUC grade 1: AUC, 0.928 (95% CI, 0.886–0.970); NUC grade 2: AUC, 0.923 (95% CI, 0.880–0.966); NUC grade 3: AUC, 0.949 (95% CI, 0.868–1.000).

**Figure 4:**
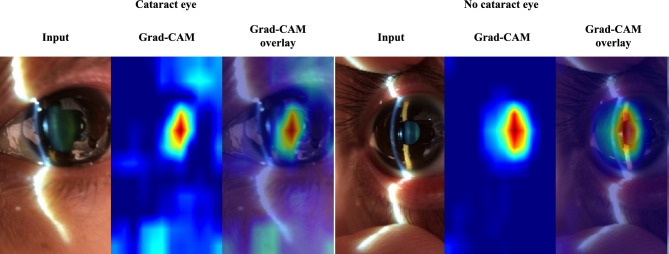
Visualization Using Grad-CAM (Gradient-weighted Class Activation Mapping): The cataract frames extracted were subjected to a post-hoc visual explanation methodology. The input data, as visualized by Grad-CAM activation mapping, produced a heatmap. Overlaying this heatmap on the input image revealed the focal region on the crystalline lens. Interestingly, for both cataract and non-cataract eyes, the model directed significant attention to the crystalline lens, as indicated by the intense heatmap.

We analyzed the performance of each NUC grading estimation of our model versus the diagnosis by ophthalmologists using the validation dataset. The NUC grading estimated by the model according to each frame was as follows: NUC grade 0: AUC, 0.961 (95% CI, 0.955–0.968); NUC grade 1: AUC, 0.910 (95% CI, 0.899–0.920); NUC grade 2: AUC, 0.903 (95% CI, 0.894–0.912); NUC grade 3: AUC, 0.901 (95% CI, 0.882–0.920) (Fig. 3).The estimation of NUC grading against the ophthalmologist accompanying each video was as follows: NUC grade 0: AUC, 0.967 (95% CI, 0.943–0.990); NUC grade 1: AUC, 0.928 (95% CI, 0.886–0.970); NUC grade 2: AUC, 0.923 (95% CI, 0.880–0.966); NUC grade 3: AUC, 0.949 (95% CI, 0.868–1.000) (Fig. 3). To visualize class activation mapping, the heatmap sufficiently overlayed the position of the crystalline lens (Fig. 4).

## Discussion

This study investigated whether the proposed ML model could diagnose NUC from videos recorded using a slit-lamp device. The diagnostic utility of the device has been demonstrated for NUC^19^. The objective was to validate the performance of the NUC diagnostic model compared with that of ophthalmologists in clinical settings^19^.

We used the following novel strategies to achieve high performance (high accuracy, sensitivity, specificity, PPV, NPV, and AUC).

- Unique data collection method: We used a portable Smart Eye Camera (SEC) as the data-collecting device because the device can record videos during the slit-lamp examination and help diagnose cataracts.
- Preprocessing before ML: The elimination of unnecessary frames, followed by an ML process to estimate the cataract grading using the extracted optimized frames.
- Rule for displaying the final result: The model estimates the NUC grading of every diagnosable frame from a single video, and the final diagnosis is determined by the majority vote for each frame.

The findings reveal that our AI algorithm exhibits excellent performance in diagnosing NUC and estimating NUC severity; the performance of the proposed model is comparable to that of ophthalmologists. To apply the model in practice, a cataract video was obtained using SEC and the data was sent to our AI model. Therefore, the final value of the model is the outcome of “mydriasis + Non mydriasis” videos.

The cataract diagnostic performance exhibited accuracy, sensitivity, specificity, and AUC of 0.942 (95%CI 0.911–0.959), 0.962 (95%CI 0.920–0.984), 0.931 (95%CI 0.905–0.944), and 0.934 (95%CI 0.897–0.970), respectively, with high PPV and NPV (Fig. 2). These values were consistent with those of previous studies demonstrating AI algorithms in cataract diagnosis (Table 1). Cheung et al., introduced a computer-aided diagnosis system utilizing slit-lamp images that achieved over 80% in both sensitivity and specificity^20,21^ (Table 1). Wu et al.^16^ demonstrated robust performance in cataract diagnostic AI using slit-lamp photographs with equal outcomes for ophthalmologists, with a sensitivity and specificity of 92.00% and 83.85%, respectively (Table 1). Jiang et al.^22^ introduced a deep learning-based lens partition model, employing multicenter datasets, which yielded accuracy, sensitivity, and specificity rates ranging from 92.57 to 97.96%, 91.95–97.04%, and 93.08–98.74%, respectively (Table 1). Focusing of different disease, Li et al.^15^ revealed a high performance in the keratitis diagnostic AI model slit-lamp and smartphone photograph (sensitivity: 81.5–98.7%, specificity: 95.0–99.8%, and accuracy: 95.4–99.3%). Zhang et al.^23^ indicated a high diagnostic accuracy range of 70.27–83.81% and 0.86–0.98 for several types of infectious keratitis (bacterial keratitis, fungal keratitis, herpes simplex keratitis, and Acanthamoeba keratitis) using a deep-learning-based diagnostic AI system. We achieved a performance similar to previous studies in which the objective findings from slit-lamp images were used.

**Table 1:** Comparison of past reference in cataract diagnostic AI.

	Year	Accuracy (95% CI)	Sensitivity (95% CI)	Specificity (95% CI)	AUC (95% CI)
Jiang et al.^22^	2021	92.57–97.96% (–)	91.95–97.04% (–)	93.08–98.74% (–)	–
Wu et al.^16^	2019	88.79% (84.88–91.98%)	92.00% (87.33–95.36%)	83.85% (76.37–89.71%)	95.96% (93.16–98.75%)
Xu et al.^21^	2013	0.690	–	–	–
Cheung et al.^20^	2011	–	81.80% (79.70–83.70)	80.70% (79.50–81.90)	0.892 (0.884–0.900)
Current study	2023	0.942 (0.911–0.959)	0.962 (0.920–0.984)	0.931 (0.905–0.944)	0.934 (0.897–0.970)

To demonstrate the high performance of our model, the following factors were considered. First, we collected a sufficiently large amount of data and performed preprocessing on the data. In a previous study, Wu et al.^16^ collected 37,638 slit-lamp photographs from 18,819 eyes to develop a cataract diagnostic AI. Li et al.^15^ acquired 13,557 anterior segment photographs to develop an AI model for screening keratitis. A small sample size results in an unstable performance of the AI models and large differences in their results^24^. Therefore, in this study, we used 38,320 frames, which was sufficient to develop an AI model as a past reference. Moreover, the rule to determine the final result as the majority consensus was another reason. Most values were improved from diagnostic performance according to the frames to the diagnostic performance according to the videos (Figs. 1 and 2).

Second, image quality issues were not observed. The training, verification, and test sets used in some AI studies are associated with image quality problems^25^, such as unclear or incomplete images in datasets^26,27^. We excluded the images of insufficient quality by setting an exclusion criteria. Moreover, we selected only diagnosable frames focusing on the crystalline lens during preprocessing. To determine the identity of the input image, we applied SEC as a data collection tool to correct the standardized anterior segment images because the angle and width of the slit light are fixed^19,28^. This standardized data collection may have contributed to the high performance of the model (Fig. 1), and the AI focused on the crystalline lens correctly by using visualization using Grad-CAM (Fig. 4). Furthermore, our observations suggest that the AI specifically focuses on the essential tissue, notably the nucleus of the crystalline lens, in both cataract and non-cataract eyes. This indicates that the algorithm does not interpret the patient's facial aging pattern but rather exclusively targets the crystalline lens, as visualized by Grad-CAM (Fig. 4). Exclusion during preprocessing and the use of a standard data-collecting device can overcome the problems associated with image quality issues.

Third, we mimicked the cataract diagnostic process performed by an ophthalmologist. In the clinical setting, ophthalmologists use a slit-lamp microscope to diagnose cataracts^29^. They visually evaluate the optical tissue and subjectively define the cataract color as integral numbers. We reproduced these diagnostic processes using SEC^19^ and ML algorithms. Moreover, our model examined each cataract video frame to arrive at a final diagnosis with a majority consensus, which may increase diagnostic precision. Generally, the development of AI for image-based diagnosis requires numerous standard images for ML. We overcame this challenge by using a diagnosable image frame and ML processes to reproduce the rationale of ophthalmologists during evaluation for a final diagnosis with a majority consensus. The high accuracy of our model can be attributed to this reason.

Fourth, simple diagnostic criteria were used. The current cataract grading system, that is, the WHO grading system, comprises three simple and easily evaluable stages^30^. The simple grading system has an excellent interobserver agreement and is used worldwide^31^. Several other cataract diagnostic criteria, such as the Lens Opacities Classification System III^32^, the Oxford Clinical Cataract Classification^33^, and the Wisconsin cataract grading^31^, are used worldwide. However, these grading systems divide NUC into several levels so that intergrader reliability is not very high (0.82–0.79)^34^. Thus, applying simple and worldwide grading may improve model performance.

This study has several limitations. First, the sample size was small. Although this study was a retrospective study in which videos were used to enhance the size of the dataset, larger datasets are required to create versatile AI models, particularly for imaging analysis. In this study, 21,306 dataset frames were used; however, the number of frames for each grade was not identical. A similar limitation of consecutive case series has been reported^9,14^. However, we minimized sample collection (only 1812 eyes) and maximized diagnostic accuracy using videos, amplifying the datasets (38,320 frames), and imitating a previous study^15^.

Second, we included cases of NUC but not of other types such as CC, ASC, and PSC. Both cataract type and severity are associated with considerable reductions in the best-corrected visual acuity^35^. However, NUCs are more common, particularly in older adults^36^, and cause visual impairment, poor depth perception, and low contrast sensitivity^29^. Further studies are needed to develop diagnostic AI for each cataract subtype, including CC, ASC, and PSC, and subsequently integrate them for a comprehensive cataract diagnosis^37^.

Finally, our dataset included data from an Asian population. Differences exist in the relative lens position and anterior chamber depth of the crystalline lens among ethnicities that exhibit variable risks of angle-closure glaucoma and/or cataract type^38^. Furthermore, our analysis was confined to test and validation datasets derived exclusively from a single medical institute. For broader clinical application, it is imperative that future studies employ external datasets to validate coagulation. However, limited studies have been conducted on the color differences in NUC among ethnicities. Moreover, our model is eligible for use in various ethnic populations; however, further validation is necessary.

Despite these limitations, the proposed ML method successfully constructed a high-performance cataract diagnostic model. To the best of our knowledge, this study is the first to investigate cataract diagnostic AI using a mobile device. Thus, the combination of a mobile device and our model could be applied to screen patients, particularly in rural and isolated areas and in disaster medicine, where ready access to ophthalmology clinics is limited. Moreover, the proposed model exhibited high sensitivity in eyes without mydriasis (“Non mydriasis” eyes) (Figs. 1 and 2). Therefore, this model can be used for patient screening during health checks and primary care.

## Materials and methods

### Ethics and information governance

This study adhered to the tenets of the Declaration of Helsinki and was conducted in compliance with the protocols approved by the Institutional Ethics Review Board of the Minamiaoyama Eye Clinic, Tokyo, Japan (IRB No. 202101). The requirement for written informed consent was waived because of the retrospective nature of the study and use of deidentified data.

### Study design

**Figure 5:**
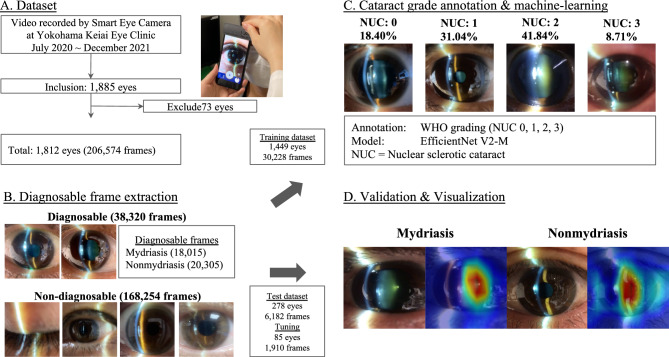
Study outline. Demographic steps of the study. (**A**) Explanation of dataset creation. (**B**) Diagnosable frame extraction. We divided all the data into diagnosable (38,320 frames) and nondiagnosable (168,254 frames). (**C**) Cataract grade annotation and machine learning. Representative images of the annotated frames. The distribution of annotations was as follows: 18.40% were classified as NUC 0, 31.04% as NUC1, 41.84% as NUC2, and 8.71% as NUC3. (**D**) Validation and visualization. Demographic images for validation.

Data were collected from a single ophthalmology institution (the Yokohama Keiai Eye Clinic). Five trained ophthalmologists used mobile video-recordable portable slit-lamp devices (details are provided in “Mobile recording slit-light device” section) to record anterior segment eye videos. All videos were recorded from July 2020 to December 2021 and assembled on a cloud server to be organized into the dataset for our study (Fig. 5). Experienced ophthalmologists utilized a mobile video-capable portable slit-lamp device to target the crystalline lens of patients using a thin slit light emitted from the device. The ophthalmologists captured anterior segment videos, consistent with those taken using conventional slit lamp microscopes. Patients were instructed to refrain from blinking during the recording, ensuring the videos mirrored those taken in typical clinical settings.

The following criteria were set for including the videos: (1) NUC videos able to diagnose using a thin slit-light beam (0.1–0.3 mm); (2) video focused into crystalline lens; and (3) video containing over 5 s of images. Other videos were excluded because they were difficult to include in this study.

A total of 1,885 Asian eyes were considered in this study. We excluded the following cases: (1) corneal diseases that adversely affected cataract grade evaluation (e.g., bullous keratopathy, corneal opacity), (2) aphakic and pseudophakic eyes, and (3) videos of insufficient quality (i.e., lack of any frame of crystalline lens). Based on these criteria, 73 eyes were excluded. In total, 1,812 eyes were included in this study. Videos containing 20,640 s (average, 19.8 s per video) were sliced at a frame rate of 60 ms and organized into 206,574 frames of static images for the dataset.

Next, we manually extracted only diagnosable frames focused on the crystalline lens. The nondiagnosable frames did not focus on crystalline lens, and the images were blurred by colored contact lenses or other noise. In total, 38,320 frames (mydriasis: 18,015 frames; and Non mydriasis: 20,305 frames) were classified as diagnosable and eligible for use.

After preprocessing to standardize the quality of each image and eliminate poor quality images and setup the datasets, 3 ophthalmologists annotated all frames at random and classified them according to the simplified cataract grading system by the World Health Organization (WHO)^30^. The distribution of annotations was as follows: 18.40% were classified as NUC 0, 31.04% as NUC1, 41.84% as NUC2, and 8.71% as NUC3 (Fig. 5). The ML process was performed on the training dataset but not on the validation dataset (details are provided in “Machine learning” section). After the NUC estimation model was developed, a valuation dataset was used to evaluate its performance.

### Mobile recording slit-light device

A Smart Eye Camera (SEC; SLM-i07/SLM-i08SE, OUI Inc., Tokyo, Japan; 13B2X10198030101/13B2X10198030201) was used to record slit-light videos as a diagnostic instrument. An SEC is a smartphone attachment that has demonstrated sufficient diagnostic function compared with conventional slit-lamp microscopes in animal^39^ and several clinical studies^19,28,40,41^. SEC mimics conventional diagnostic methods, such as slit-lamp microscopy, in the diagnosis of cataracts^19^. SEC exposes a 0.1–0.3-mm slit light sufficiently thin to observe a crystalline lens inside a nondilated and dilated pupil with a fixed angle of 45°^19^. Moreover, an SEC can record the videos of the anterior segment of the eyes; therefore, this instrument was used to record as many videos as possible to collect a large amount of cataract image data. An iPhone 7 or iPhone SE2 (Apple Inc., Cupertino, CA, USA) was used, with the resolution of the video set at 720 × 1280 to 1080 × 1920 pixels and a frame rate of 30 or 60 frames per second.

### Datasets and annotation

The dataset comprised individuals with an average age of 59.87 ± 20.91 years. Of the 1,885 eyes assessed, 902 were from male subjects and 983 from female subjects. Image distribution showed 11,243 (29.39%) were classified as no-cataract, while 27,077 (70.61%) were categorized as cataract. The video data were captured using the video application on an iPhone and subsequently stored in the MPEG-4 AAC, H.264 format as mp4 files. The analysis of these videos, including their conversion to frame images and subsequent data processing, was conducted using the OpenCV library in Python (Ver. 3.11). Annotation was performed on all diagnosable frames after preprocessing for ML and validation. Three ophthalmologists (one resident and two specialists) annotated all diagnosable frames according to the WHO cataract grading system^30^ by annotating only NUC grading to all frames but not the anterior subcapsular cataract (ASC), cortical cataract (CC), posterior subcapsular cataract (PSC), or other minor cataract types. In this study, NUC was annotated as follows: 0, none; 1, mild; 2, moderate; and 3, advanced. Frames that could not be used to diagnose NUC were omitted as noise. All annotation procedures were double blinded to avoid bias. Annotation data were averaged if the evaluation differed according to the annotator. The annotated data were graded into four levels by multiple ophthalmologists.

### Machine learning

After the preprocessing to standardize the quality of each image and eliminate poor quality images (eliminating 168,254 images), 80% of data were randomly assigned to the training dataset (1449 eyes, 30,228 frames), 5% of data were assigned to the validation dataset for hyperparameter tuning (85 eyes, 1910 frames), and the remaining data were assigned to the test dataset (278 eyes, 6182 frames). To estimate the severity of NUC using an ML model, a deep learning model was trained to output four classes using normalized images as the input. In the development of our model, we employed the libraries: PyTorch^42^, PyTorch Image Models^43^, and PyTorch Lightning^44^ for training purposes. The four classes of the output consisted of the normal eye and the three classifications of NUC (referred to as NUC 0, NUC 1, NUC 2, and NUC 3). We used a modified EfficientNet v2 (tf_efficientnetv2_m_in21ft1k) model, but changed only the final layer to a 4-class classification, and fine-tuned it using our training data^45^. The hyperparameters were set with a learning rate of 0.003, a total of 5 epochs, and a batch size of 32. For visualizing class activation mapping, we used a state-of-the-art technique, the post-hoc visual explanation method of gradient-weighted class activation mapping (Grad-CAM). Grad-CAM is a technique in computer vision to highlight the regions of interest for the prediction of a deep neural network by visualizing the gradient of the class score with respect to the image^46^.

### Statistical analysis

To compare the performance of the ML-based cataract diagnostic model with that of ophthalmologists, the accuracy, sensitivity, specificity, positive predictive value (PPV), negative predictive value (NPV), and area under the receiver operating characteristic curve (AUC) were calculated. Cataract “positive” was prescribed as NUC 1, 2, or 3, whereas cataract “negative” was prescribed as NUC 0. These performances were validated on the frame and video units because the frame video units are used to evaluate the performance of the model, and the video units evaluates the performance on a single video. We applied majority voting to display the final video decision. When the proposed ML model validated a cataract video, it sliced the video into several still images. Subsequently, the model estimated NUC gradings for each frame. Finally, the final diagnosis was determined by a majority (e.g., 100 images, NUC 0: 0; NUC 1: 25; NUC 2: 50; NUC 3: 25, classify as “NUC 2” by our model).

To analyze the performance of our model on the dilated and nondilated pupils, we analyzed each parameter for “mydriasis” and “Non mydriasis” eyes. Moreover, we analyzed the diagnostic performance of each NUC grade (0, 1, 2, and 3). Defining the target sample size is difficult because this study was an initial study of a new AI model. Therefore, we only collected data matching the inclusion criteria for a certain period. Significance tests were performed using their respective confidence intervals. Statistical analyses were performed using SPSS (ver. 25; International Business Machines Corporation, Armonk, NY, USA).

### Institutional review board statement

The study was conducted in accordance with the Declaration of Helsinki and approved by the Institutional Review Board of the Minamiaoyama Eye Clinic, Tokyo, Japan (IRB No. 202101, date of approval: 20 July 2021).

### Informed consent statement

Patient consent was waived due to the retrospective nature of the study and because we only used deidentified data. Informed consent is waived by Institutional Review Board of Minami-Aoyama eye clinic, Tokyo, Japan. Additionally, we provided opt-out documentation to give the participant a chance to refuse.

## Conclusions

The proposed cataract diagnostic AI trained using ML algorithms and video recordings acquired using a portable and recordable slit-lamp device exhibited high sensitivity and specificity for cataract diagnosis and NUC grade estimation. The performance of the proposed model was similar to that of ophthalmologists. Further investigations are necessary for validating the ability of our model to diagnose cataracts among various ethnicities and cataract types.

### Patents

OUI Inc. has the patent for the Smart Eye Camera (the publication of Japanese Patent No. 6627071). OUI Inc. also has patent pending for the algorithm (patent pending No. 2020-023514). Other relevant patent declarations relating to this study.

### Supplementary Information

Supplementary Video 1.

Supplementary Information.

## Data Availability

The data that support the findings of this study are available from the corresponding author upon reasonable request.
