# Supplementary material for: AI-based diagnosis of nuclear cataract from slit-lamp videos
Source: Sci Rep. 2023 Dec 12;13:22046. doi: 10.1038/s41598-023-49563-7 (PMC10716159; doi:10.1038/s41598-023-49563-7)
Supplement: Supplementary file 2 — Supplementary Information. [file 41598_2023_49563_MOESM2_ESM.docx]

Supplementary file

Supplementary video.

Initiate with a videographic capture sequence detailing of the right eye. Following this, isolate a high-definition still frame to serve as a reference point. Implement a Grad-CAM technique to generate a topographical heat map indicative of diagnostic focal points within the image. Superimpose this heat map onto the original frame, ensuring the region of peak intensity is congruent with the crystalline lens, specifically centered within the pupil.
